# A Comparison of Small Rodent Assemblages after a 20 Year Interval in the Alps

**DOI:** 10.3390/ani13081407

**Published:** 2023-04-19

**Authors:** Giulia Ferrari, Dino Scaravelli, Andrea Mustoni, Marco Armanini, Filippo Zibordi, Olivier Devineau, Francesca Cagnacci, Donato A. Grasso, Federico Ossi

**Affiliations:** 1 Department of Chemistry, Life Sciences and Environmental Sustainability, University of Parma, Parco Area delle Scienze 11/a, 43124 Parma, Italy; 2 Faculty of Applied Ecology, Agricultural Science and Biotechnology, Campus Evenstad, Inland Norway University of Applied Sciences, 2480 Koppang, Norway; 3 Research and Innovation Centre, Edmund Mach Foundation, Via Mach 1, 38098 San Michele all’Adige, Italy; 4 NBFC, National Biodiversity Future Center, 90133 Palermo, Italy; 5 Department of Biological, Geological, and Environmental Sciences, University of Bologna, Via Selmi 3, 40126 Bologna, Italy; 6 Research and Environmental Education, Adamello Brenta Nature Park, Via Nazionale 24, 38080 Strembo, Italy; 7 Istituto Oikos, Via Crescenzago 1, 20134 Milano, Italy

**Keywords:** assemblage composition, bank vole, Central-Eastern Italian Alps, range limits, small rodents, snow vole

## Abstract

**Simple Summary:**

The Alps are undergoing important environmental alterations. These can modify the characteristics of the habitats where several wildlife species persist, leading to a reshuffle of local assemblages (i.e., the ensemble of species in a given habitat). We investigated these aspects in alpine small rodents, monitoring their assemblage after a 20 year interval (between 1997 and 2016) in three close-by different habitats (rocky scree, grassland, and heath) at 2100 m a.s.l. elevation. We found that the bank vole, commonly considered a forest-dwelling species, was well-established in these high-alpine habitats already in 1997 and further expanded twenty years later. On the contrary, the snow vole, which is typically resident at these elevations and even above, has restricted its range across decades, being found in 2016 only in the optimal habitat for this species (rocky scree). Given the numerous factors affecting population dynamics in small rodents, it is hard to derive robust inferences from this ‘punctual’ comparison. The modification of local assemblages might be related to an ongoing modification of the local climate while also being caused by demographic processes typical of small mammal population dynamics. Long-term studies are necessary to better investigate these critical issues.

**Abstract:**

Human-induced environmental alterations in the Alps may importantly affect small mammal species, but evidence in this sense is limited. We live-trapped small rodents in the Central-Eastern Italian Alps in three close-by habitat types (rocky scree, alpine grassland, and heath) at 2100 m a.s.l. during summer-fall, in 1997 and 2016. We compared small rodent assemblages through a Redundancy Detrended Analysis (RDA). In both surveys, we detected two specialist species, i.e., the common vole (*Microtus arvalis*) and the snow vole (*Chionomys nivalis*), and, unexpectedly, the forest generalist bank vole (*Myodes glareolus*). In 1997, grassland was mainly occupied by the common vole, while the bank vole and the snow vole were sympatric in the other habitats. In 2016, the snow vole was detected only in the scree, while other species did not show distribution changes. We discuss a series of hypotheses that might have driven the differences observed across decades, among which is a species-specific response to abiotic and biotic environmental alterations, with the alpine habitat specialist moving out of sub-optimal habitats. We encourage further research on this topic, e.g., via long-term longitudinal studies.

## 1. Introduction

The alpine region is undergoing important alterations due to climate change [1,2] and, further, due to the direct impact of anthropogenic activities (e.g., [3,4]). The increase of temperatures [5], the reduction in ice and snow cover [6], the frequent extreme weather events [7], and the changes in land use consequent to the variations in the socio-economic context of the Alps [8] concur in reshaping the heterogeneity of the alpine landscape [9] and therefore of the habitats where several alpine species persist [10,11].

The ability of a given species to cope with such changes is determined by its ecological plasticity [12] or, alternatively, its adaptive capacity, via genetic evolution, to modify key life history traits that permit the survival and reproduction of individuals in an altered habitat [13]. Specifically, generalist species with a broad fundamental niche, as well as specialist ones whose specialization is reversible (e.g., dietary choice) [14], are able to track new environmental conditions [15,16], for example, expanding upslope towards new favourable areas [17,18,19]. Conversely, species whose specialization is due to physiological or morphological constraints [14] are characterized by a narrower environmental tolerance. This likely leads to low ecological plasticity and a tendency to contract their distribution range [20,21,22] or go extinct [23,24], especially when dispersal ability is limited by habitat fragmentation [25,26]. At the assemblage level, the relative occurrence of generalist and specialist species (generalist-specialist balance) depends on the suitability of the habitat for all species ensembles, i.e., considering their use of alternative resources and direct or indirect competitive interactions [27]. In the presence of competition, generalist species benefit from a lack of environmental stability and are favoured in heterogeneous and disrupted habitats, whereas specialist ones prevail in areas with high stability and a high rate of homogeneity [28]. When environmental conditions change, the relative role of species within the assemblage may therefore also shift [29], leading to biotic homogenization at the community level [29], as shown for example in a study on bird communities in the Swiss Alps [30].

The impact of modifications to the alpine environment on species assemblages can be investigated by comparing species’ responses across time (see, e.g., [30]). Despite being considered important bioindicators [18,20,31,32], studies examining such changes in the alpine small rodent assemblage are currently lacking, probably because historical data for comparison are scarcely available. Here, we relied on alpine small rodent monitoring after a 20 year interval to describe their assemblage across decades. The study was performed above the treeline in high-altitude habitats that are of particular ecological and conservation interest. In particular, these habitats are exposed to a series of climate change effects and occurring human modifications, e.g., climate-driven vegetation shifts [33], abandonment of traditional seasonal grazing practices [34], and disturbance caused by human leisure activities [35]. Notably, in the study area, a trend of temperature increase was recorded over the past 30 years, in line with what was observed throughout the alpine range (Supplementary Material S1; see also [5,36]).

**Figure 1:**
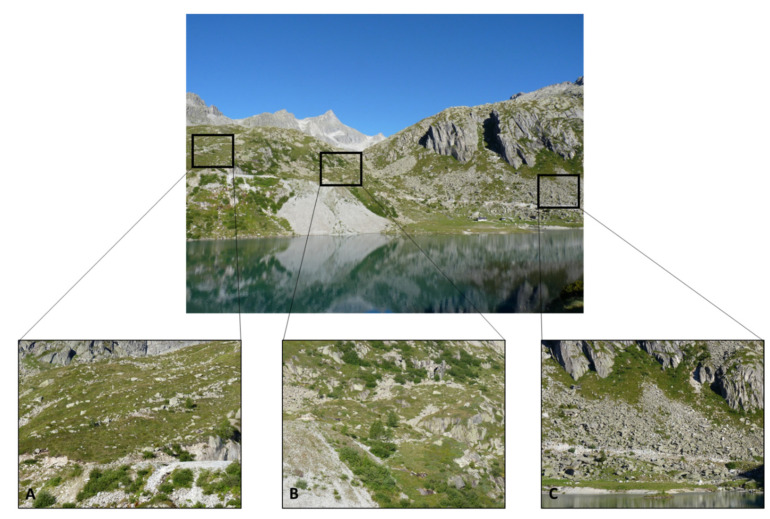
The study area and the three sampling sites (from left to right: grassland (**A**), heath (**B**), and rocky scree (**C**)).

Specifically, we carried out our descriptive comparison taking advantage of an historical study performed in 1997 on small rodents in the Central Italian Alps [37], when they were live-trapped in three grids at close-by sites with different dominant habitat types (grassland, heath, and rocky scree; Figure 1) at the same elevation. We repeated the survey in the same area with the same design for comparability after two decades (2016). Given the stochastic fluctuations characterizing population dynamics of small rodents [38], we are aware that the few intra-period (i.e., historical and current) temporal repetitions and the lack of spatial replicates limit the generalizability of results. Although the design we were able to apply does not allow us to fully address the processes underpinning variations in local assemblages, we aimed to obtain a local snapshot that can be used as a starting point for further longitudinal comparisons. At this altitudinal belt, some generalist species such as the bank vole (*Myodes glareolus*), typically considered a forest dwelling species [39,40,41] that preferentially selects old forests with dense ground cover [42,43], are at the altitudinal edge of their distribution. Conversely, habitats above the treeline are optimal for other alpine-adapted specialized species, such as the rock-dwelling snow vole (*Chyonomis nivalis*) [44]. We discuss our results in light of species functional roles and the climatic and environmental changes that have occurred.

## 2. Materials and Methods

### 2.1. Study Area and Sampling Sites

Both the 1997 and 2016 surveys were conducted in an alpine area of approximately 15,000 m^2^, placed at 2100 m a.s.l. in Nambrone Valley, in the Adamello Brenta Nature Park (46°13′24.21′′ N, 10°43′21.07′′ E, Autonomous Province of Trento, Italy). The climate of the area is typically alpine (ET class; *sensu* Köppen–Geiger classification, [45]), with cold and relatively dry winters (January: −4.5 ± 1.2 °C; 66.6 ± 80.9 mm) and a fresh and wet summer (July: 10.9 ± 1.5 °C; 129.8 ± 42.3 mm). The mean annual temperature is 2.9 ± 0.5 °C, while the mean annual precipitation is equal to 1366 ± 326 mm (data source: https://www.meteotrentino.it, accessed on 24 March 2023, Pradalago station, 6 km in line of sight from the study area; coordinates: 46°14′58.92′′ N, 10°48′50.04′′ E; altitude: 2084 m a.s.l.; data interval: 1990–2022, accessed on 12 January 2023).

Within the study area, we identified three sampling sites above the treeline, spaced 250–300 m apart and separated by microhabitat barriers (e.g., mountain streams), facing eastward and placed at the same altitude, but characterized by different habitat types (grassland, heath, and rocky scree; Figure 1), whose characteristics did not differ substantially across decades. In particular, the alpine grassland was mainly dominated by herbaceous species, with the main dominance of *Festuca* spp., *Nardus* spp., and *Carex* spp. The heath was composed of a mix of rhododendrons (*Rhododendron ferrugineum*), juniper bushes (*Juniperus* spp.), blueberries (*Vaccinium* spp.), spare mugo pines (*Pinus mugo*), and larches (*Larix decidua*). The scree area was predominantly occupied by rocks. The study area is typically inhabited by several alpine species, including small mammals and other larger herbivores such as marmots (*Marmota marmota*), alpine mountain hares (*Lepus timidus varronis*), and alpine chamois (*Rupicapra rupicapra*), while carnivores are represented by ermines (*Mustela erminea*), red foxes (*Vulpes vulpes*), and several birds of prey (e.g., *Aquila crysaetos*) [46].

### 2.2. Small Rodents Live-Trapping

In both 1997 and 2016, for each habitat type, we deployed a 256 m^2^ squared grid with 16 traps, each of which was placed at the center of a 4 × 4 m cell. In each year, we performed one session per month, from August to October. The captures lasted 6 days/5 nights, with trap controls occurring every 12 h. Before each session, we pre-baited the traps for two days to habituate individuals, filling them with apple slices and peanut butter throughout the whole capture session. In 1997, we deployed the Sherman live traps (30 × 9 × 8 cm), while in 2016, the standard Ugglan Multiple Live Traps (Model 2, Granhab, Sweden) were adopted. We performed a pilot study to account for potential differences in trapping success between the two trap models. We found a higher trapping success for Sherman traps with respect to Ugglan ones, and we took this difference into account when interpreting the results about the community composition (see below and Supplementary Material S2).

We applied the standard capture-mark-recapture (CMR) technique [47,48]. In 2016, we marked individuals by means of the fur-clipping code [49]. This non-invasive marking method allowed individual recognition in the event of recapture within the same trapping session but not among subsequent sessions due to fur re-growth. Each trapping session was therefore considered separately from the others. In 1997, animals were marked through toe clipping, a method no longer commonly accepted because of its implications for animal welfare [50], but back then adopted by the scientific community for both lab and field rodents [51] that allows individual recognition among subsequent trapping sessions. In this study, we used these old data for the sole purpose of presenting some findings of conservation interest.

At any capture event, we recorded the date, time, and trap site, as well as the species, sex, reproductive state, and age class. Specifically, we defined three age classes, following the classification provided by [52,53]: adults, including sexually mature females with opened vagina who are pregnant or lactating, and males with scrotal testes; subadults, i.e., females with inactive reproductive organs or closed vagina, and males with abdominal testes; and juveniles, where we identified sex based on the uro-genital distance, which is larger in males than females. We also recorded body mass by means of Pesola scales with an accuracy of 0.5 g, while we used a caliper (with an accuracy of 1 mm) to measure hind foot length.

According to the law regulating wildlife conservation in the Autonomous Province of Trento, none of the studied species is subject to any form of protection, and therefore no authorization is needed for capturing and handling these animals (Provincial Law 24/1992, Art. 2 [54]). However, we made our best efforts to guarantee animal welfare and minimize the risk of injury and death. Nonetheless, we recorded 7 cases of death out of 377 capture events (including also recaptures), four of which were common shrews (occasionally trapped), while the remaining were rodents (one common vole and two snow voles).

### 2.3. Statistical Analyses

We examined the small rodent assemblage across habitat types and decades by applying ordination methods for 1997 and 2016 separately. We performed these analyses relying solely on the capture data of new individuals within each session (i.e., the first capture event per individual), because the fur-clipping marking technique used in 2016 does not permit the recognition of an individual across monthly sessions due to fur regrowth.

We inspected the structure of the data by using Detrended Correspondence Analysis (DCA, [55,56]) to determine the length of the DCA first gradient. Since the length of the first DCA axis was below three standard deviation units (1.08 and 1.91, respectively, in 1997 and 2016; see Supplementary Material S3), suggesting a relatively homogenous dataset, we moved to linear methods, which are preferable in this case, and performed a Stepwise Redundancy Analyses (RDA; [57,58,59,60]), with the habitat type used as a constrained variable. For each year, we created two matrices: one for the three habitat types and the other for species occurrence and abundance in each of the habitats. We considered the data collected in the three trapping sessions of each year separately because the interval between sessions was long enough for new individuals to occur, either newborns or dispersers. We linearly combined the two matrices to maximize habitat separation between species, reporting the overall and axis-related proportion of variance explained by the habitat type as well as the canonical coefficients (CC) [58,61]. Monte Carlo permutation tests were used to assess the significance of constraints (999 permutations; [62]). Lastly, we represented the association between small rodent species and habitat type by means of ordination biplots [63].

We performed the analyses using R software 4.1.2 [64] and specifically the packages *ggvegan* [65], *vegan* [65], and *tidyverse* [66].

## 3. Results

Overall, we captured 67 individuals in 1997 and 51 individuals in 2016. In both surveys, we detected the bank vole (*Myodes glareolus*), the common vole (*Microtus arvalis*), and the snow vole (*Chionomys nivalis*) (Table 1; for details about the number of individuals per habitat type, age class, and sex across decades, see Supplementary Material S4). We also occasionally captured the common shrew (*Sorex araneus*) and the alpine shrew (*Sorex alpinus*), but we did not include these species in any analysis because the study design was not fitted to monitor these species. The common vole was found in grassland and heath but not in the scree, both in 1997 and 2016, while the bank vole was recorded in all three habitats across decades. The snow vole occurred in all three habitats in 1997 but not in 2016, when it was found in the scree only. In the snow vole, body mass was measured on average at 37.7 ± 9.1 g, with individuals being heavier in 2016 (42.9 ± 6.7 g) than in 1997 (35.2 ± 9.1 g). The bank vole and the common vole weighed on average 23.5 ± 5.3 g and 21.8 ± 3.6 g, respectively, with no major differences denoted across decades (see Supplementary Material S5 for details).

**Figure 2:**
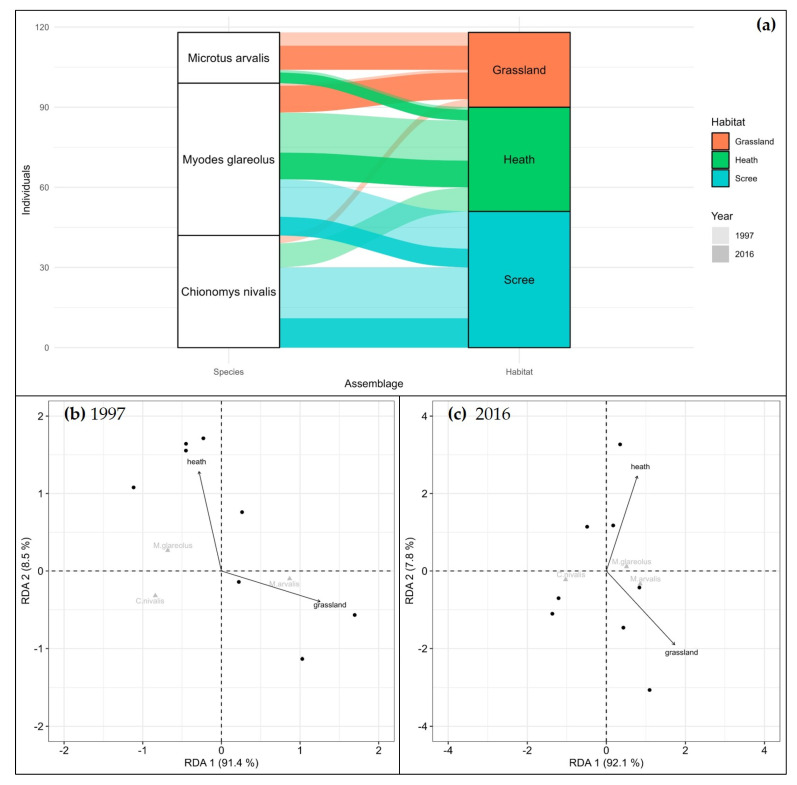
(**a**) Alluvial plot visualizing frequency distributions of small rodent species across habitat types in 1997 and 2016. (**b**,**c**) An RDA biplot showing the association between small rodents and habitat types in 1997 (**b**) and 2016 (**c**).

When occurrences of these species were represented as assemblages, variations were mainly observed in the grassland and in the heath, where the snow vole disappeared from 1997 to 2016, leading to an increment in the relative frequencies of the common vole and the bank vole (Figure 2a). In the scree, the composition of the assemblage did not undergo apparent modifications across decades.

The association between habitat types and species changed across decades. In 1997, the biplot showed a slight dissociation of the snow vole and the bank vole from the grassland on the first axis, while the common vole was not associated with a specific habitat. To a lesser extent, the second axis dissociated the bank vole, occupying the heath, from the snow vole, which was more associated with the scree habitat (Figure 2b). Yet, this association between small rodent abundance and habitat type was not significant (F_2,66_ = 2.26, *p* > 0.05). In 2016, instead, the snow vole was markedly separated from grassland on the first axis. The second axis separated the common vole in the grassland from the bank vole, which did not exhibit any association with a given habitat (Figure 2c). The observed association between small rodent abundance and habitat type was statistically significant (F_2,50_ = 2.49, *p* = 0.03).

Specifically, the RDA analysis evidenced that habitat type accounted for almost 43% of the variance in small rodent assemblage composition in 1997 and around 46% in 2016. In particular, the RDA first axis explained 39% and 42% of the overall explained variance in 1997 and 2016, respectively. Both in 1997 and in 2016, this axis was positively associated with grassland (CC = 0.95 and CC = 0.67, respectively). The second axis, which in both analyses explained a much lower percentage of variance (4% and 3% in 1997 and 2016, respectively), was positively associated with heath in both surveys (1997: CC = 0.98; 2016: CC = 0.95) (Supplementary Material S3). In 1997, the first axis clearly delineated a separation between the snow vole (CC = −0.84) and the bank vole (CC = −0.68) on its negative side, from the common vole (CC = 0.87). In 2016, the first axis separated only the snow vole (CC = −1.03) from the other species, which were all positively related to the first axis, although to a different extent (in decreasing order of absolute value of variance: common vole (CC = 0.85), bank vole (CC = 0.51)). With respect to the second axis, the overall separation pattern was less evident. In 1997, it separated the snow vole (CC = −0.32) from the bank vole (CC = 0.26) in the heath, while the common vole was scarcely associated with the second axis. In 2016, the common vole was negatively associated with the second axis (CC = −0.33), as were the snow voles (CC = −0.22), while the bank vole (CC = 0.11) was slightly and positively associated with the second axis (Supplementary Material S3).

## 4. Discussion

This study represents, to the best of our knowledge, the first inter-decadal comparison of the small rodent assemblage at the margin of treeline in the Alps. The bank vole was well-established in this area above the treeline, both in 1997 and in 2016, showing a partially unexpected plasticity in adapting to more open habitats with respect to its preferred forest ([43]). Conversely, the snow vole was present in all habitats in 1997, but it was not detected in grassland or heath after two decades, when it was the most associated species with scree. This meant an overall decrease in the association between these two small rodents after a 20 year interval. In this study, we cannot disentangle which proximate mechanism operated, given the simple design and the lack of independence between data gathered at the same site through time. On the one hand, it is possible that the fast-ongoing environmental modifications, and in particular the warmer weather, may force the snow vole to move towards its most preferred habitat, i.e., the scree, where the species can find heat shelters. Alternatively, it is possible that demographic outbreaks that occurred in the past may have caused, in 1997, an expansion of snow vole populations towards suboptimal habitats. The mechanisms behind assemblage shifts also include the effects of abiotic changes and consequential variations in habitat suitability on species interactions, such as competition between species. Further comparisons across altitudinal and latitudinal gradients may help investigate the generality and mechanisms of these observed effects. It has also to be noted that the absence of data about the snow vole in the heath and grassland in 2016 could partly be explained by the different traps used (Supplementary Material S2). However, the change in numbers of captured individuals is abrupt, and at least part of the detected change is likely genuine, thus allowing a comparison of the assemblage of the species.

Although occasionally recorded even up to 2700 m a.s.l. in the Alps, the bank vole is typically considered a species living below the timberline [67]. Our findings suggest that this species, at least in the study area where we performed the survey, was well established above the treeline in open areas. Although recorded in both surveys, the species was slightly dissociated from grassland in 1997, while it was not associated with any habitat type in particular in 2016. The average body mass of adult bank voles in the study area is close to the lower end of the known body mass distribution [67], which suggests that these habitats might be marginal for the species persistence [68]. With the data at our disposal, it is hard to infer the processes underpinning the colonization process by this forest-dwelling small rodent at these altitudes. Under the assumption that in an alpine environment a temperature increment can lead to a resource distribution modification [69,70], we may speculate that the altitudinal habitat suitability for the bank vole increased over time. Therefore, this plastic species might benefit from these favourable environmental changes, as observed for other montane species worldwide [31]. The well-known trend towards milder climate observed in the Central-Eastern Alps ([36]; see also Supplementary Material S1), combined with the great thermal tolerance and plasticity observed in the bank vole [71,72,73], may support this view, which however needs to be confirmed by further analysis based on long-term time series of demographic studies. Indeed, we cannot definitively exclude that both in 1997 and in 2016, the species was present in this marginal area because of density-dependent dispersion towards sub-optimal habitats, as previously argued [74,75].

The common vole has already been reported to typically dwell in alpine grassland [76]. Our results confirm the prevalent association of this species with this habitat type, and the morphometric measures that we report fit with what is known about this species [67]. The observed co-occurrence of the common vole and the bank vole in the grassland and, to a minor extent, in the heath is likely permitted by a niche separation driven by food strategy differentiation (the bank vole is an omnivore, while the common vole diet is strictly herbivorous, including roots and leaves; [41]).

The most evident modification of assemblage composition after 20 years was the dissociation of the snow vole from other species, with this rock-dwelling specialist isolating itself in its optimal habitat [34]. A possible explanation for what was observed, which however remains speculative, is that the snow vole might have moved towards the scree to contrast increasing temperature. Hence, microhabitat conditions might favour the persistence of this specialist species [44], which is characterized by a narrow thermal tolerance [77]. This interpretation finds some support in the growing body of evidence about behavioural adaptations that several alpine species are developing to cope with heat stress (see e.g., [78] for a case study on the Alpine ibex, *Capra ibex*). The increment in temperature in the study area might have had a double-sided effect on the snow vole, restricting its tolerance to more extreme alpine habitats while ameliorating the individual conditions therein (see Supplementary Material S4 and S5). Indeed, snow vole body mass, whose values were aligned with what is known for this species [67], increased in 2016, possibly reflecting an increment in resource availability [79]. However, our results do not permit us to clearly understand the intrinsic mechanisms underpinning such changes. As an alternative hypothesis, it is possible that the snow vole population in 1997 expanded towards grassland and heath in consequence of stochastic demographic outbreaks, which are characteristic of small mammal population dynamics [38]. Abrupt increases in population abundance during outbreak years may have produced density-dependent emigration of dispersers towards marginal habitat for the species [80]. Lastly, the ongoing expansion and establishment of the bank vole in the area might have pushed the snow vole out of less extreme habitats, where the bank vole likely thrives. Yet, there is no evidence for competition between these two species [77], which, according to the data at our disposal, co-occur in the scree.

## 5. Conclusions

Our results present some empirical support for a habitat shift for the snow vole and for some shifts in the small rodent assemblage in general. Further work should focus on disentangling the process of association/isolation of species from other species-specific processes such as density-driven expansions/contractions induced by demographic outbreaks [81] or climate-driven behavioural adaptations [82].

Despite being aware that through our comparison we cannot infer the proximate causes of small rodent assemblage variation, we maintain that this work constitutes a starting point to investigate in this direction. The evidence for global and climate change is ubiquitous [2], and the Alps are among the areas on the planet where these modifications are occurring faster [83], as reflected in different types of species responses. For instance, the alpine marmot’s (*Marmota marmota*) litter size decreased over a 20 year time scale in relation to climate change effects [84], while recent empirical work proves that the altitudinal limit of the hazel dormouse (*Muscardinus avellanarius*) is higher than expected [85]. It is of the utmost importance to strengthen this evidence by analyzing the effect of environmental changes on community and population dynamics of alpine species. This can be obtained via long-term comparisons in the same monitored areas or, alternatively, by applying space-for-time substitution approaches such as an assessment along a latitudinal or altitudinal gradient [86,87]. This research line is particularly recommended in those areas where the coexistence of generalist and specialist species occurs in ecosystems with “nowhere to go”, such as the alpine ones.

## Figures and Tables

**Table 1:** Contingencies of the three rodent species across years and habitat types.

Species	1997			2016		
	Grassland	Heath	Scree	Grassland	Heath	Scree
*Chionomys nivalis*	3	9	19	-	-	11
*Myodes glareolus*	1	15	14	10	10	7
*Microtus arvalis*	5	1	-	9	4	-

## Data Availability

The data presented in this study are available as Supplemental Material.

## Supplementary Materials

The following supporting information can be downloaded at: https://www.mdpi.com/article/10.3390/ani13081407/s1: Supplementary Material S1: Temperature trends in the study area; Supplementary Material S2: Trapping success comparison between Ugglan and Sherman traps; Supplementary Material S3: Ordination Analysis; Supplementary Material S4: Visualization of captured individuals in function of habitat type, age class, and sex across decades; Supplementary Material S5: Assessment of variation in individual morphometric measures across decades; Supplementary Material S6: Comparison of body mass in snow voles in the scree habitat between 1997 and 2016. References [88,89,90] are in Supplementary Materials.
