# Supplementary material for: A Comparison of Small Rodent Assemblages after a 20 Year Interval in the Alps
Source: Animals (Basel). 2023 Apr 19;13(8):1407. doi: 10.3390/ani13081407 (PMC10135415; doi:10.3390/ani13081407)
Supplement: Supplementary file 1 [file animals-13-01407-s001.zip › animals-2251434-supplementary.pdf]

Supplementary Materials

Supplementary Material S1: Temperature trends in the study area

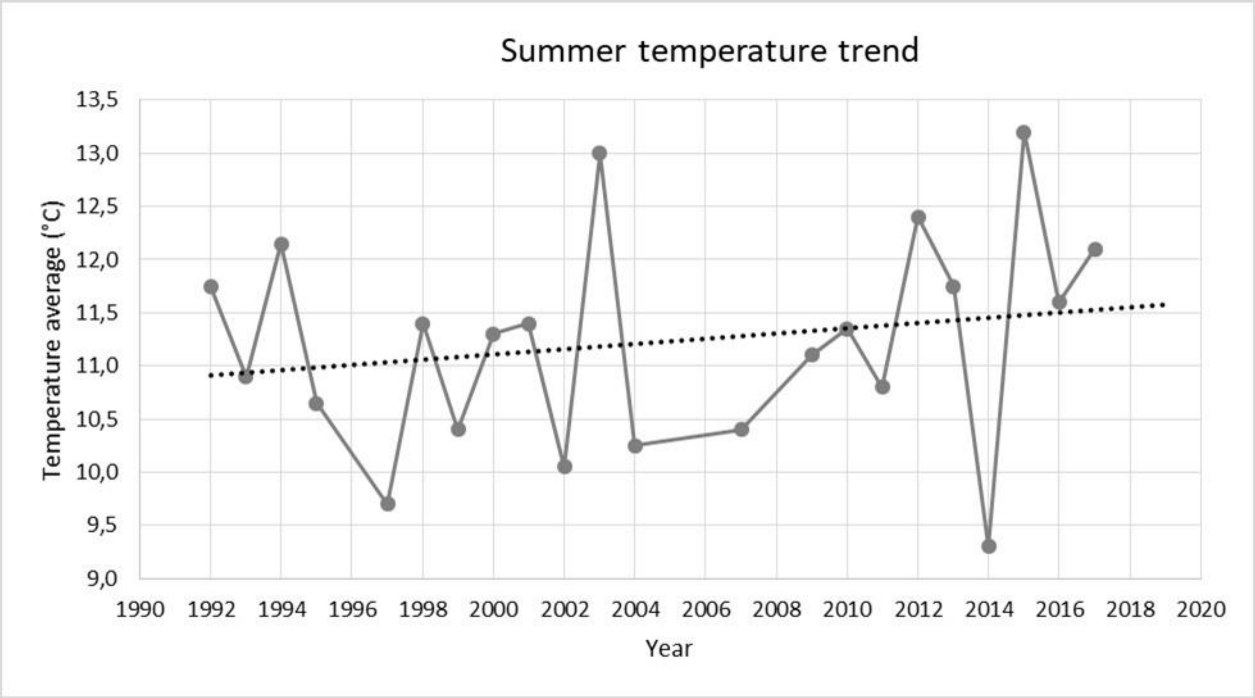

Figure S1.1. Trend of mean temperature in summer (July and August) from 1992 to 2017 (Data source: www.meteotrentino.it, Pradalago station; coordinates: 46°14'58.92"N, 10°48'50.04"E; altitude: 2084 m a.s.l.).

Supplementary Material S2: Trapping success comparison between Ugglan and Sherman traps

The trap model implemented for the survey differed between 1997 and 2016. While in 1997 we used the Sherman live traps (30 x 9 x 8 cm), in 2016 we adopted Ugglan traps (24 x 8 x 6 cm). Since the trap model can influence the capture success [1–3], in 2016 we performed a pilot study to test whether the trapping success differed between the two models. To this end, in the scree habitat we deployed a grid of 10 Sherman live traps at 200-300m distance from the Ugglan grid made of 16 traps. We performed two capture sessions in September and October, checking the traps for 6 days/5 nights every 12 hours, for a total of 9 trap controls. We used a Generalized Linear Model with Poisson distribution to model the number of first captures per session in function of the species and the trap model, while accounting for the exposure (i.e., trap-controls).
We found that trapping success with Ugglan traps was significantly lower to that of Sherman live traps (β = -0.69 ± 0.18; $p < 0.001$; Figure S1.1). Moreover, trapping success was higher for snow vole than bank vole (β = 0.69 ± 0.19; $p < 0.001$; Figure S1.1), which were the only two species detected (Table S1.1). It is unlikely that the differential trapping success was due to local differences in abundance of these two rodent species, since the two trapping grids were relatively close (200- 300 m apart) and deployed in sites where environmental conditions potentially affecting small rodent abundance did not differ (e.g., resource availability, predators presence). We thus conclude that Sherman live traps, at least in our study system, allow a higher capture success than Ugglan ones. The higher trapping success for snow vole than bank vole is likely related to the habitat where this comparison has been performed, i.e., the scree that is the optimal habitat for snow vole but less for bank vole.
Despite the differential trapping success between the two models, we retain that the differences detected between 1997 and 2016 small rodent assemblage composition cannot be imputed to such a methodological issue. For example, the abrupt increase of bank vole detected in the grassland in 2016 (10 individuals vs 1 in 1997) is contradictory with what one would have expected if the differences were driven by the trapping success between the two trap models. Even for what concerns snow vole, the lack of occurrence of this species in the heath and grassland in 2016 cannot be solely explained by the trap model, since the species has been captured with Ugglan traps in the scree habitat. Other ecological processes underpin the observed changes, that we discuss in the main text.

Table S2.1. Contingency table of first capture events (FCE), for each species and trap model. The number of trap controls is reported.

Species	FCE	Trap_model	Trap Controls
Myodes_glareolus	3	Ugglan	180
Myodes_glareolus	5	Sherman	288
Chionomys_nivalis	5	Ugglan	160
Chionomys_nivalis	11	Sherman	100

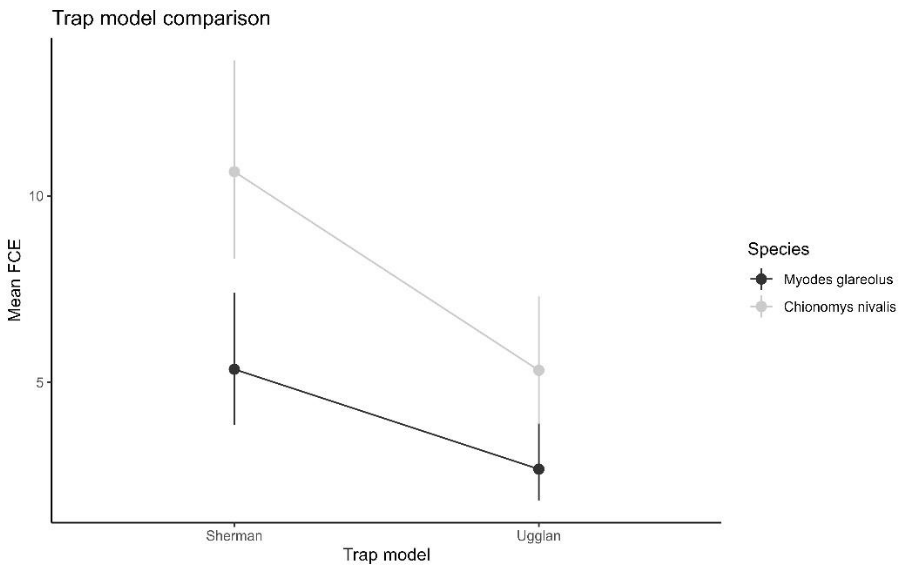

Figure S2.1. Contrast analysis showing the differential capture success between trap models and rodent species (bank vole, *Myodes glareolus*; snow vole, *Chionomyis nivalis*)

Supplementary Material S3: Ordination analysis

We report the results of the DCA and RDA analyses, for 1997 and 2016 surveys.

Table S3.1. Eigen values and gradient length from the Detrended Correspondence Analysis (DCA) in 1997.

	**DCA1**	**DCA2**	**DCA3**	**DCA4**
**Eigen values**	0.64	0.30	0.36	0.38
**Decorana values**	0.65	0.08	0.007	0.003
**Axis length**	1.08	0.82	0.73	0.74

Table S3.2. Summary of the RDA of the small rodent assemblage in 1997, with respect to the habitat typology.

**RDA Total variance**	**Constrained**	**Unconstrained**	**Explained variation**		
3.00	1.29	1.71	42.94%		
	**RDA1**	**RDA2**	**PC1**	**PC2**	**PC3**
**Eigen values**	1.18	0.11	0.77	0.53	0.41
**Proportion explained**	0.39	0.04	0.26	0.18	0.14
	**Canonical Coefficients (CC)**				
**Constraining variables**	**RDA1**	**RDA2**			
grassland	0.95	-0.30			
heath	-0.22	0.98			
**Species scores**	**RDA1**	**RDA2**	**PC1**	**PC2**	**PC3**
Snow vole	-0.84	-0.32	-0.53	0.33	-0.66
Common vole	0.87	-0.10	0.26	0.87	0.23
Bank vole	-0.68	0.26	0.95	0.05	-0.43

Table S3.3. Eigen values and gradient length from the Detrended Correspondence Analysis (DCA) in 2016.

	**DCA1**	**DCA2**	**DCA3**	**DCA4**
**Eigen values**	0.57	0.18	0.18	$1.84e^{-01}$
**Decorana values**	0.61	0.00	0.00	$6.49e^{-05}$
**Axis length**	1.91	1.03	1.03	$1.02e^{+00}$

Table S3.4. Summary of the RDA of the small rodent assemblage in 2016, with respect to the habitat typology.

**RDA**<br>**Total variance**	**Constrained**	**Unconstrained**	**Explained variation**		
3.00	1.36	1.64	45.45%		
	**RDA1**	**RDA2**	**PC1**	**PC2**	**PC3**
**Eigen values**	1.26	0.11	1.00	0.41	0.22
**Proportion explained**	0.42	0.03	0.33	0.14	0.07
	**Canonical Coefficients (CC)**				
**Constraining variables**	**RDA1**	**RDA2**			
grassland	0.67	-0.74			
heath	0.30	0.95			
**Species scores**	**RDA1**	**RDA2**	**PC1**	**PC2**	**PC3**
Snow vole	-1.03	-0.22	0.32	-0.42	0.49
Common vole	0.85	-0.33	-0.52	-0.68	-0.23
Bank vole	0.51	0.11	1.12	-0.19	-0.25

Supplementary Material S4: Visualization of captured individuals in function of habitat type, age class and sex across decades

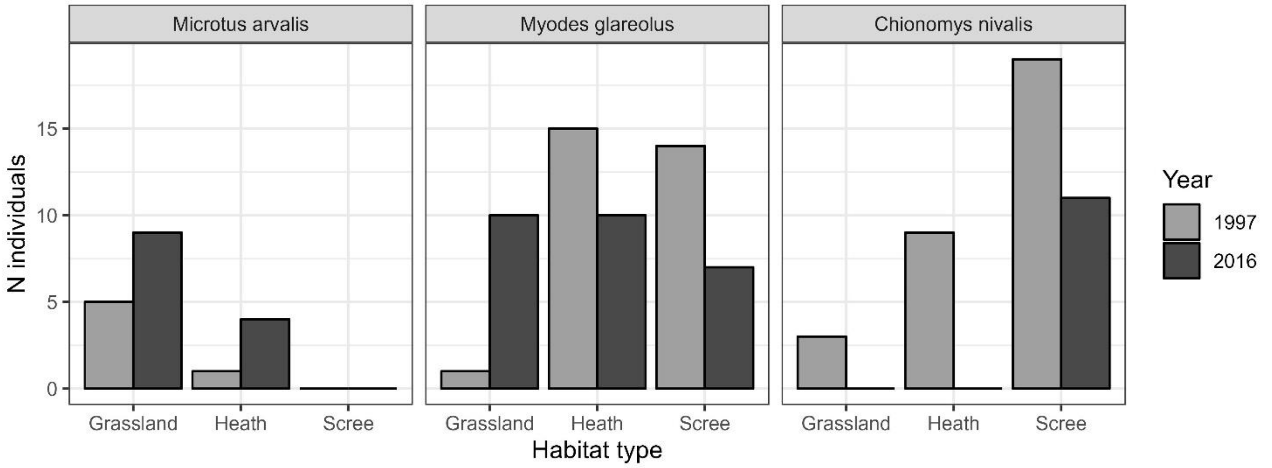

Figure S4.1. Barplot showing the number of individuals captured in 1997 (grey) and 2016 (black) in the three habitat types (grassland, heath, rocky scree) and across species (*Microtus arvalis, Myodes glareolus, Chionomys nivalis*).

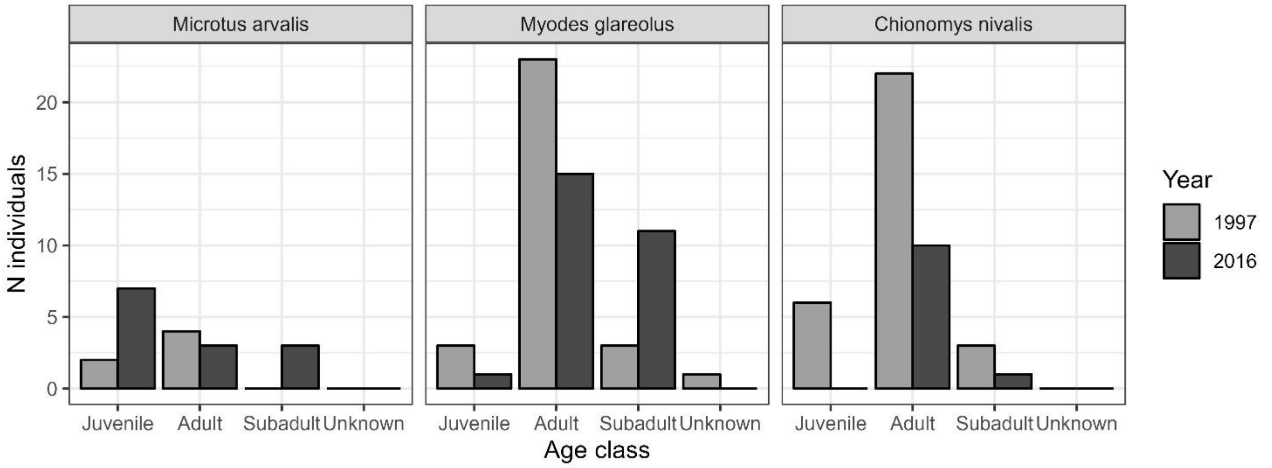

Figure S4.2. Barplot showing the number of individuals captured in 1997 (grey) and 2016 (black) depending on age class (Juvenile, Adult, Subadult, Unknown) across species (*Microtus arvalis, Myodes glareolus, Chionomys nivalis*).

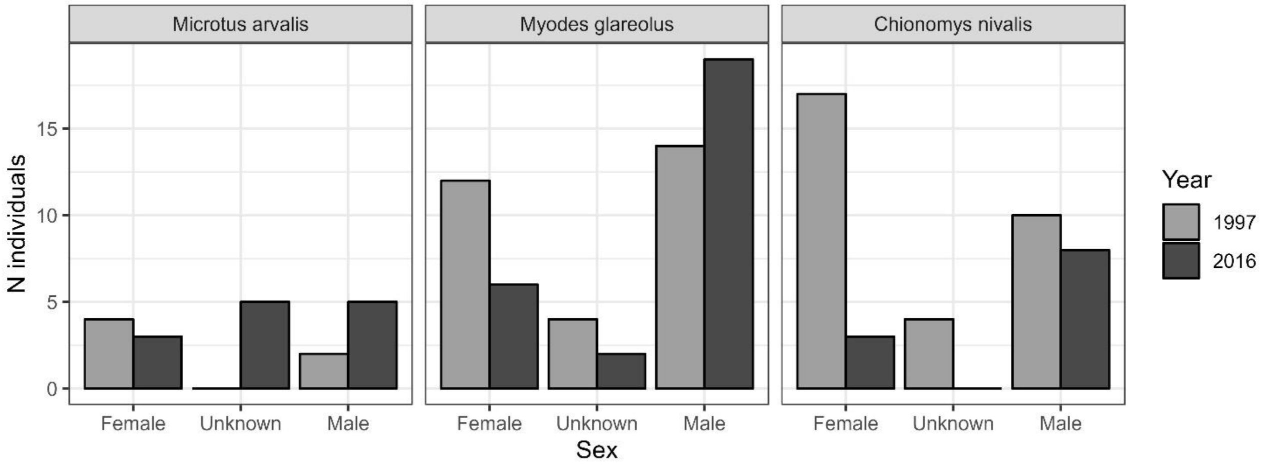

Figure S4.3. Barplot showing the number of individuals captured in 1997 (grey) and 2016 (black) depending on sex (Female, Male, Unknown) across species (*Microtus arvalis, Myodes glareolus, Chionomys nivalis*).

Supplementary Material S5: Assessment of variation in individual morphometric measures across decades

In parallel with the empirical comparison of small rodent assemblage, we investigated inter-decadal variation in individual morphometric traits comparing body mass and hind foot length of small rodents across decades. We limited this analysis only to adults and subadults, for which morphometric data had been collected. For each species and trait, we tested differences between 1997 and 2016, grouping all the data collected across the three habitats, and fitting a Student's t-test or a Mann-Whitney-Wilcoxon non-parametric test when the assumptions were violated.

In snow vole, body mass was significantly lighter in 1997 (M = 35.17 g, SD = 9.06 g) than in 2016 (M = 42.86 g, SD = 6.74 g) (t(25.93) = -2.77, p = 0.01), while hind foot length did not differ significantly across decades. In bank vole, none of the morphometric traits differed between 1997 and 2016 (body mass in 1997: M = 23.81 g, SD = 5.10 g; in 2016: M = 23.12 g, SD = 5.69 g). Common vole body mass did not change across decades (1997: M = 24.37 g, SD = 2.93 g; 2016: M = 20.16 g, SD = 3.12 g), while hind foot length decreased significantly from 1997 to 2016 (t(7.54) = 2.96, p = 0.01). These results are illustrated in the Figure S5.1.

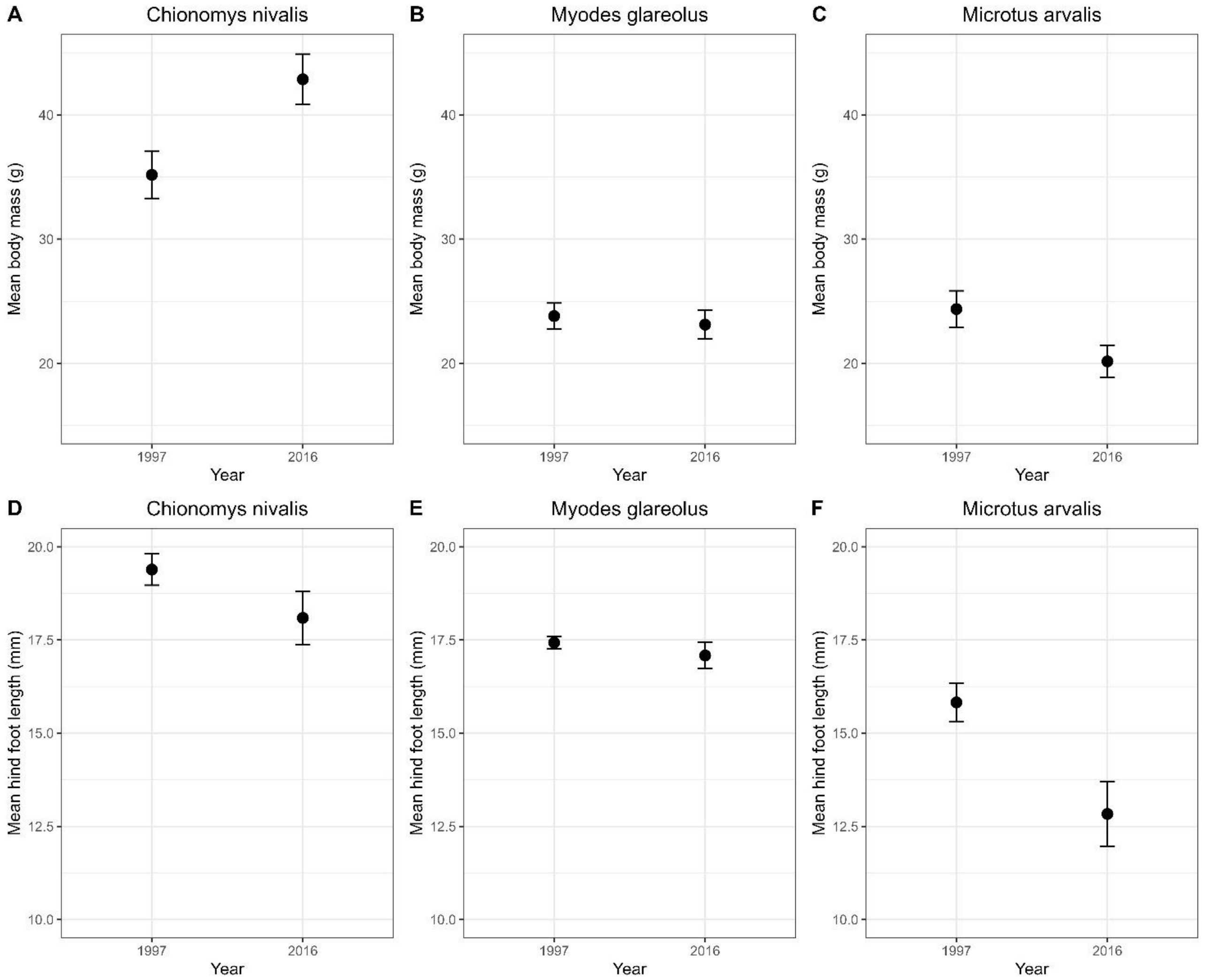

Figure S5.1. Morphometric measures (body mass and hind foot length) for *Chionomys nivalis, Myodes glareolus* and *Microtus arvalis* across decades. The dot identifies the mean value; the bars denote the standard error.

Supplementary Material S6: Comparison of body mass in snow vole in the scree habitat between 1997 and 2016

The detected difference of body mass in snow vole between 1997 and 2016 might have been driven by a difference in occurrence of the species in the surveyed habitats (see Table 1 and Figure 2 in the main text). To check whether this was the case, we performed the same comparison, but limited to the scree habitat, where the species had been detected both in 1997 and 2016. Body mass (t(23.95) = -3.00, p = 0.006) increased between 1997 and 2016 (Figure S5.1), confirming the observed general pattern. This result highlights that the difference in body mass across decades was not linked with the typology of habitat where the species occurred. The interpretation of this finding is presented in the discussion of the main text.

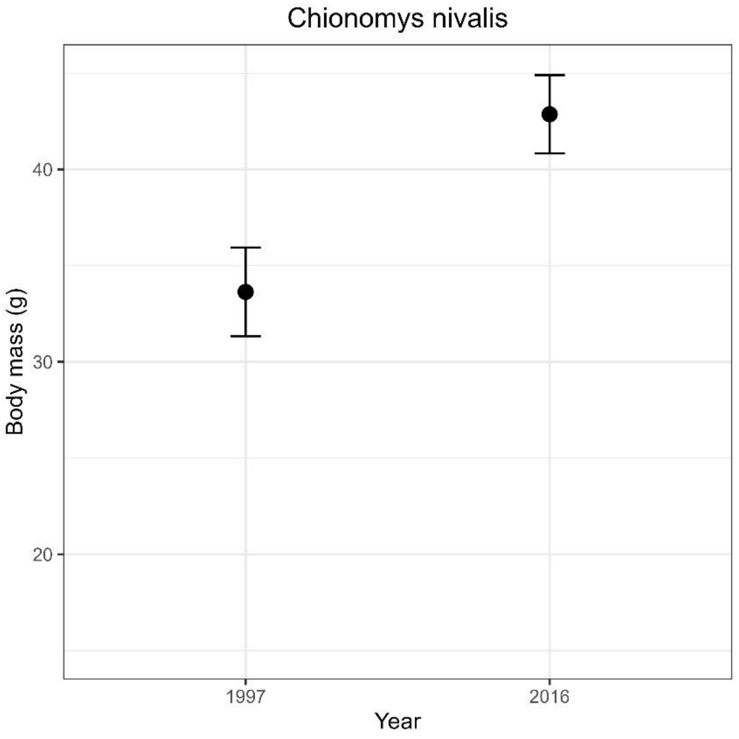

Figure S6.1. Plot of body mass of snow vole solely in the scree habitat in 1997 and 2016. The dot identifies the mean value; the bars denote the standard error.

References

1. O'Farrell, M.J.; Clark, W.A.; Emmerson, F.H.; Juarez, S.M.; Kay, F.R.; O'Farrell, T.M.; Goodlett, T.Y. Use of a Mesh Live Trap for Small Mammals: Are Results from Sherman Live Traps Deceptive? *J. Mammal.* **1994**, *75*, 692–699, doi:10.2307/1382517.
2. Ylönen, H.; Jacob, J.; Kotler, B.P. Trappability of rodents in single-capture and multiple-capture traps in arid and open environments: why don't Ugglan traps work? *Ann. Zool. Fennici* **2003**, *40*, 537–541.
3. Jung, T.S. Comparative efficacy of Longworth, Sherman, and Ugglan live-traps for capturing small mammals in the Nearctic boreal forest. *Mammal Res.* **2016**, *61*, 57–64, doi:10.1007/s13364-015-0251-z.
